# Adaptive coupling influences generalization of sensorimotor learning

**DOI:** 10.1371/journal.pone.0207482

**Published:** 2018-11-29

**Authors:** Mohsen Sadeghi, James N. Ingram, Daniel M. Wolpert

**Affiliations:** 1 Department of Engineering, University of Cambridge, Cambridge, United Kingdom; 2 Zuckerman Mind Brain Behavior Institute, Department of Neuroscience, Columbia University, New York, United States of America; University of California Merced, UNITED STATES

## Abstract

Sensorimotor learning typically shows generalization from one context to another. Models of sensorimotor learning characterize this with a fixed generalization function that couples learning between contexts. Here we examine whether such coupling is indeed fixed or changes with experience. We examine the interaction between motor memories for novel dynamics during reciprocating, back and forth reaching movements. Subjects first experienced a force field for one movement direction and we used channel trials to assess generalization on the reciprocal movements. This showed minimal coupling such that errors experienced for one movement direction did not lead to adaptation for the other. However, after subjects had experienced a force field for both movement directions concurrently, a coupling developed between the corresponding motor memories. That is, on re-exposure for one direction there was a significant adaptation for movements in the other direction. The coupling was specific to the errors experienced, with minimal coupling when the errors had the opposite sign to those experienced during adaptation. We developed a state-space model in which the states for the two movement directions are represented by separate, yet potentially coupled learning processes. The coupling in the model controlled the extent to which each learning process was updated by the errors experienced on the other movement direction. We show that the coupling relies on a memory trace of the consecutive errors experienced for both movement directions. Our results suggest that the generalization of motor learning is an adaptive process, reflecting the relation between errors experienced across different movements.

## Introduction

An important feature of sensorimotor learning is the degree of generalization (or transfer) from one context to another. For example, when learning a visuomotor rotation [1–6] or a dynamic force-field perturbation [7–14], there is limited transfer of learning from one movement direction to another. Many current models of sensorimotor learning use state-space models in which a context-dependent generalization function quantifies the amount of the transfer between the various contexts [6, 7, 9, 14–20]. Such generalization functions are often modelled as a Gaussian, centred on the current context (the movement direction or the orientation of a hand-held object, for example), and selectively weight the influence of errors when updating the states in the model. As such, error-dependent learning is greatest for states associated with the current context, and progressively reduces for more distant contexts. These models, however, assume that the generalization function is fixed and independent of experience. In the current study, we directly test this assumption by examining the interaction between motor memories when subjects adapt to force-field perturbations in a reciprocating reaching task.

Reciprocating movements consist of back and forth movements, where, the dynamics associated with each movement may be the same or different. For example, when rowing or swimming, the movement of the oar or hand consists of two components. The first component (the power stroke) is performed under the water, thrusting the boat and body forward. The second component (the recovery stroke) is performed above the water to return the oar or the arm back to its original position for the next cycle. Each movement component is performed in a different environmental condition (in this case, under versus above the water) and with different dynamics applied to the movement. Similar reciprocating movements can be associated with tool use, such as using a saw or a wrench, which also have different dynamics for each component of the movement. In such cases, separate motor memories may be required to implement appropriate motor commands for each movement. In other examples of reciprocating movements, both movement components can have similar dynamics, such as erasing a whiteboard or polishing a surface. In these cases, a consistent dynamic model can be applied to perform both movements.

In the current study, we examined whether the coupling of learning between two given contexts (movement components) is not fixed, but rather adapts depending on the experienced dynamics in each context, with increased coupling when the movements share the same dynamics. We regard this coupling as a form of generalization as it reflects how adaptation generalizes between movements in opposite directions (i.e., 180 degree generalization). We conducted experiments in which subjects performed reciprocating (back and forth) reaching movements between two targets. We manipulated the dynamics of the reaches using force-field perturbations in one movement, and tracked how adaptation changed in the reciprocal movement. This allowed us to examine the extent to which the corresponding motor memories were coupled, and how the coupling changed under various dynamic conditions. We found that coupling increased when errors were experienced in both movement directions concurrently, leading to enhanced generalization of learning from one movement direction to another. However, when different dynamics were experienced for the two movements, minimal coupling developed. To account for these results, we developed a novel state-space model in which an adaptive process mediates the coupling between the learning processes associated with each movement direction.

## Materials and methods

Fifty subjects (29 female; mean±sd age: 26.9±3.9 years) with no known neurological disorders, provided informed written consent and participated in the experiment. All subjects were right handed according to the Edinburgh handedness inventory [21] and were naive to the purpose of the experiment. The protocol was approved by the University of Cambridge Psychology Research Ethics Committee.

Experiments were performed using a vBOT planar robotic manipulandum, with associated virtual reality system and air table [22]. The vBOT is a custom-built back-drivable planar robotic manipulandum exhibiting low mass at its handle. The position of the vBOT handle is calculated from optical encoders and force at the handle is controlled by specifying torque at the motor (both at 1 kHz). Subjects grasped the handle of the vBOT with their right hand, with their forearm supported by an air sled which constrained movement to the horizontal plane. Visual feedback was provided using a computer monitor mounted above the vBOT and projected into the plane of movement via a mirror. This allowed us to display targets and a cursor representing hand position (targets and cursor were 0.5 cm radius disks).

**Fig 1:**
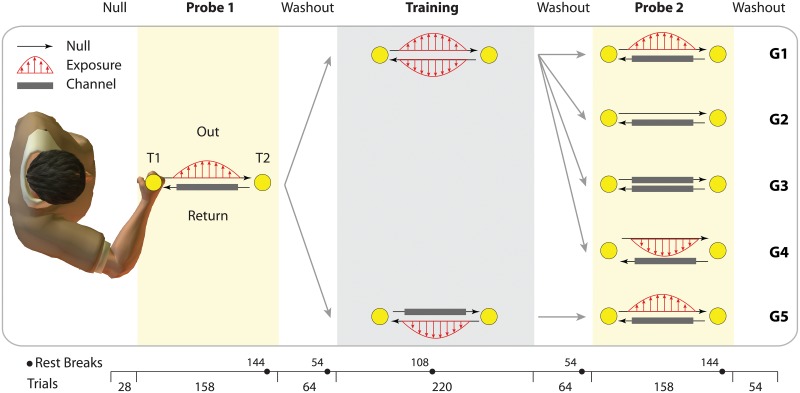
Experimental paradigm. Subjects made Out and Return reaching movements between two targets (T1 and T2), while in each movement direction, one of three trial types (dynamics) was introduced: exposure to a velocity-dependent force field, channel trials, and null trials (see legend). The experiment consisted of three main phases: Probe1, Training and Probe2. In Probe1, all groups experienced a force field on the Out movement, and channels on the Return. This allowed us to measure the *a priori* coupling (transfer of learning) between the Out and Return movements. For the Training phase, subjects in G1 to G4 experienced exposure in both Out and Return movements concurrently, whereas for G5, subjects only experienced force field for Return movements. This was to test the effect of concurrent versus successive exposure on the coupling between Out and Return movements. The five groups further differed in Probe2 as illustrated. Channel trials were interspersed in all phases. Each phase was followed by a block of null trials (the “washout”). The number of trials in each phase is shown at the bottom of the figure. Subjects received multiple rest breaks during the experiment, indicated by the black circles (the numbers next to each circle indicates the trial number, withing the given block, after which the rest break was introduced). The paradigm was counter-balanced across subjects within each group, both for the direction of the Out movement (T1 to T2, or T2 to T1) and the force field direction (clockwise or counterclockwise).

The task required subjects make reaching movements back and forth between two targets (T1 and T2 in Fig 1). The targets were in the mid-sagittal plane and 12 cm apart. Across pairs of trials, subjects would first reach from one target to the other (e.g., T1 to T2), and would then reach back to the first target (T2 to T1). The first movement of the pair was referred to as the Out movement. The second movement of the pair was referred to as the Return movement. The targets (and therefore movement directions) for the Out and Return movements were counter-balanced across subjects within each experiment. At the start of each trial, subjects were required to keep the cursor stationary (with velocity less than 0.5 cm/s for 100 ms) within the start target for that movement (which could be either T1 or T2, as described above). The reach target then appeared on the screen and a tone cued the subject to make a reaching movement to that target. Subjects had to initiate the movement within one second of the tone, otherwise a mistrial was triggered and the trial repeated. They were also encouraged to finish the trial (be within the reach target for 100 ms with velocity less than 0.5 cm/s) within 800 ms, or a warning message “Too slow” was displayed. The next trial started after 500 ms so that Out and Return movements were clearly separate movements. Note that, due to counter-balancing described above, an Out movement was made away from the body for half the subjects and towards the body for the other half.

### Trial types

Using the vBOT, we could impose various dynamics on the subject’s arm during the reaches. We used three different trial types; null trials, exposure trials, and channel trials. In a null trial, the vBOT generated no forces. In exposure trials, the vBOT generated a velocity dependent curl-field in which a force was applied on the subject’s hand. The force direction was orthogonal to the movement direction. The force magnitude depended on the movement velocity. Specifically, the force was given by:

$$\begin{array}{c} \begin{array}{c} \left[\begin{array}{c} F_{x}\\ F_{y} \end{array}]=b[\begin{array}{cc} 0 & 1\\ -1 & 0 \end{array}][\begin{array}{c} \dot{x}\\ \dot{y} \end{array}\right] \end{array} \end{array}$$ (1)

where, *F*_*x*_ and *F*_*y*_ were the components of the force, $\dot{x}$ and $\dot{y}$ were the components of hand velocity, and the constant *b* determined the gain and direction of the field, with *b* = 15 Ns/m for a clockwise (CW) field and *b* = −15 Ns/m for a counter-clockwise (CCW) field. In a channel trial, a mechanical channel was applied which constrained the hand movement to a straight line (within a few millimetres of lateral displacement) between the two targets by simulating a stiff one dimensional spring and damper (spring coefficient, 6 kN/m; damping coefficient, 5 Ns/m). This is a measure of feed-forward adaptation to the force field [23, 24].

We manipulated the trial type separately for Out and Return movements. In some trials, both movements were of the same trial type (i.e., both null, both exposure, or both channel), and in others, separate trial types were used for each movements (e.g., exposure for Out and channel for Return).

### Experimental design

The aim of the study was to investigate the coupling (transfer) of learning processes between reciprocal (Out and Return) movements. We focused on two main questions in the designing of each experiment. First, whether there was any *a priori* coupling between Out and Return movements. Second, whether the coupling could change, depended on the dynamics experienced across repeated pairs of Out and Return movements.

Each experimental group performed three main phases. First, the initial probe phase (Probe1), in which we measured the *a priori* coupling. In this phase all Out movements were performed in a force field and all Return movements were performed in a channel (Fig 1, Probe1). This allowed us to examine how learning in the Out movements transferred to the Return movements. Second, in the Training phase, subjects experienced the force field on both movement directions (G1 to G4) or only on the Return movement (G5) with channels on the Out movements (Fig 1, Training). Finally, a second probe phase (Probe2) was used in which we examined coupling under the various dynamic manipulations of the task (Fig 1, Probe2). After each phase (Probe1, Training and Probe2), subjects performed a block of null-field trials (washout) to return adaption to the baseline.

Subjects were randomly assigned to one of five groups (n = 10 each group). All groups underwent a familiarization session in which they performed 40 null trials in both movement directions. These trials were not analyzed. The experiments started with 14 pairs of Out-Return null trials followed by the three phases (Probe1, Training and Probe2). During Probe1, all groups of subjects performed 79 pairs of Out-Return movements (158 trials in total), in which subjects adapted to the force field exposure during Out movements (65 exposure trials with 14 channel trials at pseudorandom locations), while the Return movements were all channel trials (Fig 1, Probe1). This phase was followed by a short washout block consisting of 30 pairs of null trials followed by 2 pairs of channel trials (64 trials in total).

In the Training phase, subjects from G1 to G4 were exposed to the force-field perturbation for both Out and Return movements. This phase consisted of 90 pairs of exposure trials (in both Out and Return directions) with 20 pairs of channel trials pseudo-randomly placed in between (two pairs of channel trials were always placed at the end of the phase and the other 18 pairs were interspersed between the exposure trials with the ratio of 5 to 1). In G5, subjects only experienced the force field in the Return movements, while the Out movements were all channel trials (Fig 1, G5). Importantly, this group experienced force fields for both Out and Return movements, but in separate phases of the experiment and never concurrently. This allowed us to contrast the effects of concurrent force-field exposure experienced by G1 to G4. The Training phase was followed by a short washout block, as described above.

Finally, Probe2 was used to examine the interaction of Out and Return movements after the Training phase and was identical to the Probe1, except for the dynamics in Out movements (the Return movements were always performed in a channel). Specifically, the Out movement could be either the same force field as in the Training phase (G1 and G5), null trials (G2), channel trials (G3), or a force-field opposite to the Training phase force field (G4).

In each group, the force field direction during the experiment was counter-balanced between the subjects, such that half the subjects experienced CW force field on exposure trials, and the other half experienced CCW force field. The results from these subgroups were then appropriately combined for analysis by flipping signs of the position/force data (note that for G4, subjects who experienced CW force field for Probe1 and Training phase, were exposed to CCW force field for the Probe2 phase, and vice versa). All subjects received rest breaks (90 seconds) at fixed intervals during the experiment (Fig 1). The 10 trials that immediately followed a rest break were excluded from the analysis. For Probe2, all the trails following the break were excluded (14 trials).

### Analyses and statistical tests

We recorded the position and velocity of the hand and forces produced by the vBOT at 1 kHz. We used two measures of performance. On null and exposure trials, we measured the maximum perpendicular error (MPE) of the hand from the straight line between the two targets. On channel trials, we measured adaptation by regressing (with no intercept) the time course of the force that subjects produced into the channel wall, against the ideal force profile that would fully compensate for the force field. The regression included a window of data points for each trial, which began when the hand velocity first exceeded 5% of its maximum value, and ended when the velocity reduced to less than 5% of its maximum value. We used the velocity along the channel to predict the force the vBOT would have applied on an exposure trial. The slope of this regression gave a measure of adaptation (force compensation), with 0 indicating no compensation and 1 indicating perfect compensation.

The across-group statistical tests were performed using one-way ANOVA. For multiple comparisons between groups (that is, separately comparing G1 with each of the other groups), the Tukey–Kramer method [25] was applied to correct for family-wise error rate due to multiple comparisons. This was performed on Matlab (R2017b, The MathWorks Inc., Natick, MA, USA) using commands *anova1* (for across group comparisons) and *multcompare* (for corrected pair-wise comparisons) in conjunction. Prior to every statistical test, the Grubbs’ criterion [26] was applied to detect and remove potential outlier data points from the analysis. This was implemented using *isoutlier* command in Matlab. The outcome of the Grubbs’ test is only reported when an outlier is found. All the *p*-values are presented with the potential outliers removed, unless otherwise specified. To test the significance of quantities withing each group the two-tailed student t-test was used.

## Results

We investigated the generalization of adaptation (coupling) between consecutive movements in a reciprocating movement task. Subjects grasped the handle of a robotic manipulandum and performed reaching movements back and forth between two targets (T1 and T2; Fig 1). The dynamics of the movement could be separately manipulated on Out (odd numbered) and Return (even numbered) trials. Each trial could be one of three types (see Methods for details). On null trials, the robot generated no force. On exposure trials, the robot generated a velocity-dependent force field (with either a clockwise or counter-clockwise field direction). On channel trials, the robot constrained the movement to a straight line to the target.

Each experiment consisted of three main phases (Fig 1): an initial probe phase (Probe1), a Training phase, and a second probe phase (Probe2). Each phase was followed by a period of null trials (to return any adaptation back to baseline). We calculated two performance measures. On null and exposure trials, we measured the maximum perpendicular error (MPE) of the hand trajectory from the straight line to the target. On channel trials, we measured the adaptation index as the regression coefficient between the force generated by the subject into the channel wall and the ideal force that would fully compensate for the force field.

Five groups of subjects participated in the experiment and each group was exposed to different task dynamics (G1 to G5; Fig 1). Importantly, all groups experienced the same dynamics for Return trials across the different phases of the experiment: channel trials during both Probe1 and Probe2, and exposure trials during the Training phase. The groups differed in the dynamics experienced for the Out trials in each phase.

### Baseline coupling

**Fig 2:**
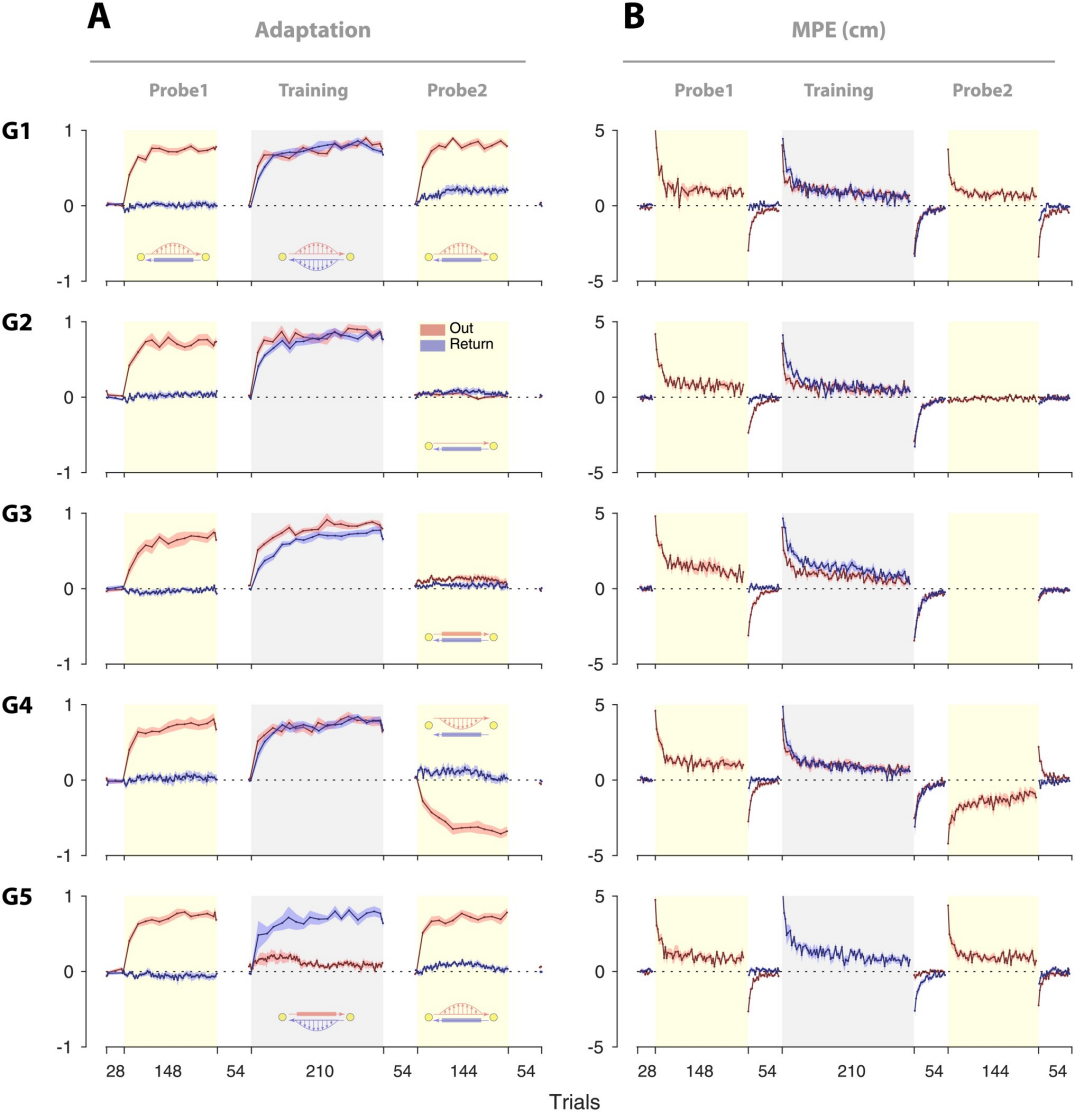
Performance across the three phases for the five groups. (**A**) Adaptation and (**B**) kinematic error (MPE) shown for each group (G1 to G5). Plots are mean ± s.e. across subjects for Out (red) and Return (blue) movements. The sign of the adaptation is relative to the force field experienced during training so that positive values correspond to appropriate compensation for the Training field. Similarly, MPE is positive for errors that are in the same direction as the Training force field. Note that the negative adaptation and MPE for the Out trials of Probe2, G4, reflects adaptation to the opposite force field, relative to Training.

Fig 2 illustrates the overall performance in terms of adaptation and MPE for all groups. The sign of the adaptation is relative to the force field experienced during Training so that positive values correspond to appropriate compensation for the Training force field. Similarly, MPE is positive for errors that are in the same direction as the Training force field.

In Probe1, all groups performed reciprocating movements with exposure to the force field during Out movements and with all Return movements in a channel. This allowed us to assess the degree to which adaptation in one movement direction (Out) transferred to the other (Return). Across the trials of Probe1, the adaptation for Out movements (measured on occasional channel trials) increased (red lines, Fig 2A), and the MPE (measured on exposure trials) decreased progressively (red lines, Fig 2B). In contrast, the adaptation measured on Return movements (blue lines Fig 2A) remained close to baseline. To quantify the final learning we averaged the adaptation for the channel trials in the last 12 movements of each direction (that is, all channel trials for the Return and 2 channel trials for the Out movements). The adaptation for the Out movement reached an average of 0.71–0.77 by the end of Probe1 across the groups (Fig 2A, Probe1, red lines) with a significant increase from 0 (t-test, *t*(9)>11.14, *p* < 0.0001 for each group) but no significant difference across the groups (one-way ANOVA showed no main effect of group: *F*_4,45_ = 0.278, *p* = 0.891). The adaptation in Return movements (ranging -0.06 to 0.04 across the groups) showed no significant difference from 0 (t-test: |*t*(9)| < 1.66, *p* > 0.13 for all groups), and did not differ significantly across groups (one-way ANOVA: *F*_4,45_ = 1.047, *p* = 0.393). This indicated minimal transfer of adaptation from the Out movements to the Return movements, implying that the sensorimotor processes associated with Out and Return movements were independent in Probe1. Subjects then performed a series of null trials to return any adaptation to baseline (“washout” phase).

### Changes in coupling with experience

We next examined whether the absence of coupling observed in Probe1 could be altered by learning consistent dynamics for both the Out and Return movements. To this end, the first 4 groups (G1 to G4) performed a Training phase in which they were exposed to a force field for both movement directions. Learning was observed for both directions, with adaptation increasing and MPE decreasing progressively throughout the Training phase (Fig 2, G1 to G4). In these groups, the adaptation for the Out movements in the Training phase appears to rise relatively faster than the adaptation for the Return movements (adaptation in the first channel trial of the Training phase was significantly larger for the Out movement compared to the Return movement; paired t-test: *t*(9) > 2.26 and *p* < 0.05 for all group). This could be due to the prior experience of a force field for the Out movement (i.e., during Probe1), which led to faster relearning during the Training phase (i.e., the Savings phenomenon [27, 28]).

A fifth group (G5) was also exposed to a force field during the Return movements of the Training phase. Unlike the other groups, however, this group experienced channel trials during the Out movements. Thus, in contrast to the other groups, G5 did not experience the force field consistently on both Out and Return movements. By the end of the Training phase, the adaptation on the Return movements was at a similar level for all groups (ranging 0.73–0.81; one-way ANOVA: *F*_4,45_ = 0.976, *p* = 0.43). All groups then experienced a series of null trials to washout any adaptation. Importantly, by the end of the washout phase, adaptation for the Return movements was again close to baseline for all groups (ranging -0.003–0.03 for the last channel trial of the washout; one-way ANOVA: *F*_4,45_ = 0.677, *p* = 0.61). Therefore, all groups exhibited a similar level of adaptation during the Training phase and had returned to baseline before starting the Probe2 phase.

**Fig 3:**
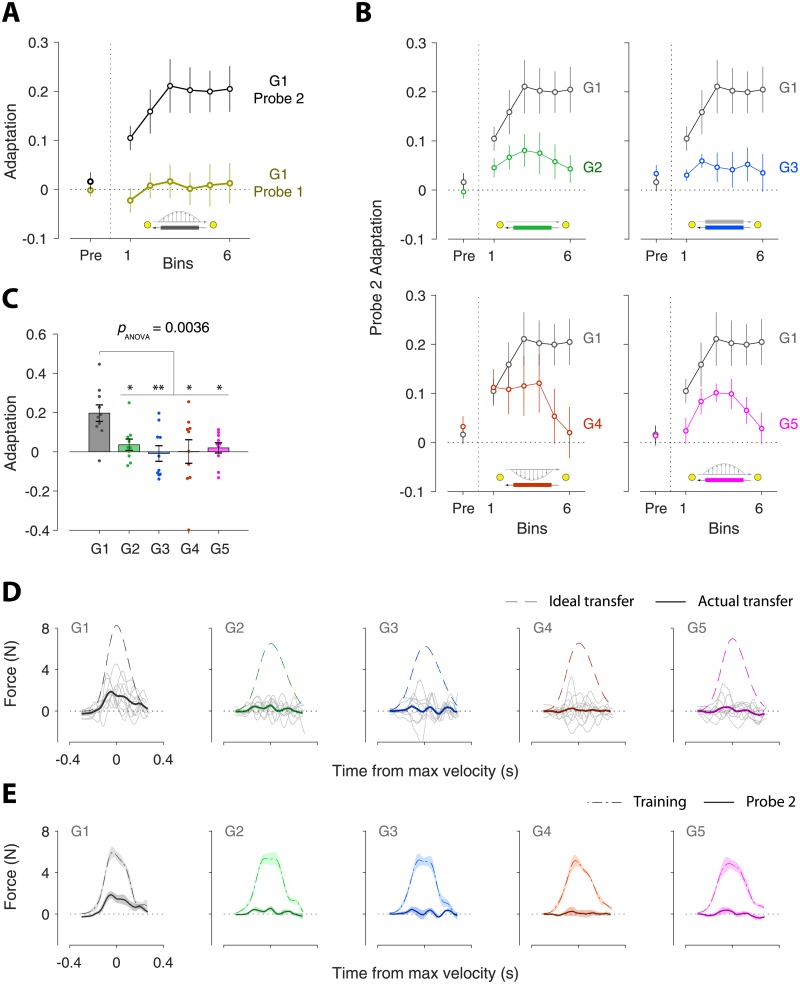
Adaptation during Probe2 for the five groups. **A**. Adaptation for the Return trials of Probe2 for G1 (mean ± s.e. across subjects in 6 bins of 12 trials each). Probe1 is shown for comparison. The Pre data point shows the last channel trial of the preceding null field (“washout”) phase. **B**. Adaptation for Return trials of Probe2 for G2 to G5. For comparison the Probe2 phase of G1 is also shown (black), plotted as in **A**. **C**. The baseline-corrected adaptation (mean ± s.e.) for the last bin of Probe2 for each group. This is obtained by subtracting the baseline adaptation (i.e., ‘Pre’ in panel **B**) from the adaptation of the final bin. The small dots represent the individual data. The *p*-values reflect the Tukey-Kramer corrected ANOVA between G1 and each of the other groups (*p < 0.05, **p < 0.01; see Results for details). **D**. The time course of generated force against the channel during the last trial bin in Probe2, shown for all groups. The trajectories are baseline-corrected and aligned based on the time at the maximum velocity during the movement (time 0). The dashed line represents the ideal force profile for full compensation, and the solid lines show the actual force profile generated by subjects (the faint lines indicate the individual force trajectories, with the mean trajectory superposed). **E**. Similar to **D**, the force trajectories for the final bin in Probe2 phase (solid line) are compared to the force trajectories at the last 5 channel trials of the Training phase (dash-dot line) for the Return movements (shaded area shows the s.e. across subjects). The force trajectory for G1 shows that the induced force due to coupling (Probe2) is similar to the previously learned trajectory (Training) but scaled down in magnitude.

The first group performed a Probe2 which was identical to Probe1, with force field exposure on Out movements and channels on Return movements (Fig 1, G1). This allowed us to examine whether the Training phase affected the coupling of adaptation for Out and Return movements. In contrast to Probe1, in which adaptation in Return trials remained at baseline, adaptation increased across Probe2 for G1 (Fig 2A, G1, blue line). Fig 3A shows the adaptation for G1 in both the Probe1 and Probe2 phases (binned in 6 time periods, with 12 trials for each bin). In both cases, adaptation prior to each probe phase (i.e., the preceding washout period) was at the baseline level (Fig 3A, Pre), with no significant difference between Probe1 and Probe2 (paired t-test: *t*(9) = 0.76, *p* = 0.47). However, by the final time period (last trial bin), adaptation in Probe2 was significantly larger than adaptation in Probe1 (paired t-test: *t*(9) = 5.85, *p* = 0.0002). We hypothesized that this increase in adaptation was due to the development of a coupling between the Out and Return movements during the Training phase. Under this coupling hypothesis, experience of the force field on consecutive Out and Return movements leads to enhanced generalization of learning, so that experiencing the force field for the Out movements in Probe2 leads to increase in adaptation for the Return movements.

### Coupling versus spontaneous recovery

An alternative explanation for the putative coupling observed in G1, is that the increase in adaptation in Probe2 is a result of spontaneous recovery of the adaptation on the Return movements. Indeed, it has been shown that under certain conditions, when rapid de-adaptation is followed by channel trials, adaptation shows a rebound (increase) due to spontaneous recovery [28–30]. To examine whether spontaneous recovery underlies the increase in adaptation, in a second group (Fig 1, G2) we replaced the force-field trials in the Out movements of Probe2 with null trials. This ensures that any increase in adaptation could not be due to coupling. In this group, spontaneous recovery appeared as a small rebound in adaptation on the Return movements, which reached up to 0.08±0.033 (mean±s.e.; paired t-test between the highest adaptation in Probe2 and the preceding washout: *t*(9) = 2.74, *p* = 0.023). However, by the end of Probe2, the adaption was not significantly different from zero (Fig 3B, G2; mean±s.e. of adaptation in the final bin 0.043±0.028; *t*(9) = 1.72, *p* = 0.12).

In order for across-group comparisons of Probe2 adaptation, we first corrected for the baseline adaptation in each group. That is, the adaptation in the preceding washout phase (the ‘Pre’ adaptation in Fig 3B) was subtracted from probe2 adaptation for each subject. This corrected for potentially different starting points for adaptation at the beginning of Probe2 phase for each group. Comparing the baseline-corrected adaptation between G1 and G2, we found that the overall adaptation (averaged across all trial bins) was significantly larger for G1 compared to G2 (Tukey-Kramer corrected ANOVA showed main effect of group: *p* = 0.0297; see Methods for details on statistical test). Importantly, the decay of adaptation in Probe2 was delayed for G1, such that adaptation in the final bin was larger for G1 compared to G2, with a difference close to significance (Fig 3C; Tukey-Kramer corrected ANOVA: *p* = 0.059). A Grubbs’ test (see Methods) showed a potential outlier for G2 (the individual with the largest adaptation; Fig 3C, G2), which was then excluded from the analysis. After repeating the test, G1 showed a significantly larger adaptation for the final bin compared to G2, indicating a longer persistence of adaptation during Probe2 for G1 (Tukey-Kramer corrected ANOVA: *p* = 0.022). This suggests that spontaneous recovery is not the only contributor to the increased adaptation observed in G1.

Because G2 experienced null trials on the Out movements, it is possible that coupling to these null dynamics could reduce the spontaneous recovery observed on the Return movements. We excluded this possibility with a third group of subjects who experienced channel trials on both the Out and Return movements of Probe2 (Fig 1, G3). Adaptation on the Return movements during Probe2 in this group was very similar to G2, with no significant increase (Fig 3B, G3; maximum increase of adaptation from baseline: 0.059±0.014; paired t-test with the preceding washout level: *t*(9) = −1.17, *p* = 0.272). As with G2, baseline-corrected overall adaptation in Probe2 (averaged across all trial bins) was significantly larger for G1 compared to G3 (Tukey-Kramer corrected ANOVA: *p* = 0.0014). Similarly, the final level of adaptation in G3 was significantly smaller than that of G1, indicating a prolonged increase of adaptation for G1 (Tukey-Kramer corrected ANOVA for the final bin: *p* = 0.0064). Together, the results from G2 and G3 suggest that although spontaneous recovery contributes to the adaptation rebound to a certain extent, other factors beyond spontaneous recovery account for the adaptation increase observed in G1.

### Specificity in coupling

To further examine whether the coupling developed in G1 was specific to the force field experienced during the Training phase, a fourth group, like G1, performed Out movements in a field in Probe2. However, in this case, the direction of the field was reversed relative to the Training phase (Fig 1, G4). In this group, adaptation in Out movements increased in the negative direction, indicating adaptation to the reversed (opposite) force field (Fig 2A, G4, red line). If the coupling was non-specific, any adaptation transferred to the Return movements would also be in the negative direction. However, negative adaptation was not observed in the Return movements. Fig 3B (G4), shows the Return adaptation index of G4 in 6 time periods (red line). As can be seen, adaptation in G4 shows a rebound in the positive direction, returning to the baseline by the end of the phase. This is inconsistent with the non-specific coupling between adaptation in Out and Return movements. In addition, when comparing the degree of adaptation between G4 and G1, although the overall baseline-corrected adaptation (averaged across all bins) was not significantly different between the two groups (Tukey-Kramer corrected ANOVA: *p* = 0.235), adaptation was preserved longer towards the end of the phase for G1 (Fig 3C; Tukey-Kramer corrected ANOVA on the final bin: *p* = 0.011). Further, the rebound of adaptation in G4 was similar to that of G2 and G3 in the final bin with no main effect of group (Tukey-Kramer corrected ANOVA between G4 and G2: *p* = 0.979; and between G4 and G3: *p* = 0.983). These results indicate that the rebound of adaptation in G4 could be due to spontaneous recovery, without the influence of coupling. This suggests that the coupling which develops during the Training phase is specific to the force field experienced in this phase.

### Coupling due to concurrent adaptation

Finally, we assessed whether paired experience of the force field in both movement directions during the Training phase in G1 was necessary for the development of coupling. One alternative hypothesis might be that, when a memory of the force field is formed for each movement direction, concurrently or in separate phases (i.e., Probe1 and Training), re-exposure to errors in Probe2 for the Out movements could reactivate the memory for the Return movements. To rule out this alternative, we examined a fifth group (Fig 1, G5). This group, like the other groups, experienced the force field on both Out and Return movements, however, in separate phases. Specifically, they experienced the field on Out movements of Probe1 (with channels on Return movements), and experienced the field on Return movements during the Training phase (with channels on Out movements). The Probe2 phase in this group was identical to G1. Fig 2A (G5) and Fig 3B (bottom right panel) illustrate the adaptation of Return movements in Probe2. The results show a rebound in adaptation, reaching up to 0.1, and decaying back to baseline by the end of the phase. This behaviour was qualitatively different from the coupled adaptation observed in G1, both in the peak magnitude and the persistence by the end of the phase (Fig 3B). The overall baseline-corrected adaptation (averaged across bins) was larger for G1 during Probe2 compared to G5, although the significance was not found (Tukey-Kramer corrected ANOVA: *p* = 0.1497). However, comparing the adaptation in the final bin, G1 showed a significantly larger level, indicating a longer lasting adaptation in this group (Tukey-Kramer corrected ANOVA between G1 and G5 at the final bin: *p* = 0.0251). These results indicate that the induced adaptation observed in G1 requires coupling between the Out and Return movements via concurrent exposure to the force field in both movement directions.

Overall, we found a clear group effect on the final level of adaptation in Probe2 (baseline-corrected last bin; one-way ANOVA across all groups: *F*_4,44_ = 4.574, *p* = 0.0036), showing that the persistence of adaptation during Probe2 was modulated by the experience of force field in the Training phase, as well as the dynamics experienced in the Out movements of the Probe2 phase. We further examined the force trajectories generated on final-bin channel trials of Probe2 to examine whether the observed adaptation (particularly for G1: ∼ 0.2; Fig 3B) was, indeed, due to velocity-dependent force generation. Fig 3D illustrates the ideal (dashed line) and actual (solid line) force trajectories for the final bin of Probe2 for each group. The trajectories were baseline-corrected (based on the force trajectories in the preceding washout phase), and were aligned according to the time of maximum velocity during the movement. For each group, force trajectories were first averaged across the channel trials of the last bin for each individual (faint grey lines). The outcome was then averaged across individuals for that group (superposed thick line). As shown, while the generated force trajectories were almost flat for G2 to G5, G1 shows a clear, bell-shaped velocity-dependent force trajectory that is similar to the ideal force profile in shape, while scaled down in magnitude. This bell-shaped force profile indicates that the observed final adaptation in G1 is likely due to the transfer of velocity dependent force-filed from the Out movements to the Return movements.

We further compared the force trajectory of Probe2 (solid lines), with the force generated during the last 5 channel trials of the Training phase (dash-dot lines) for each group (Fig 3E). This was to examine any qualitative difference between the induced force trajectories in Probe2 and that of previous learning in the Training phase. As shown, the force trajectory of Probe2 for G1 was similar to that of the Training phase, but scaled down in magnitude. Particularly, no significant difference was found for the timing of the peak force (paired t-test: *t*(9) = 1.57, *p* = 0.150), or the timing of the force onset (i.e., when force exceeded 5% of its maximum value; paired t-test: *t*(9) = 0.57, *p* = 0.580) between Probe2 and the Training phase.

### Adaptive coupling model

We first provide a brief overview of the model before describing the details. The model posits that the Out and Return movements are represented by separate adaptive processes, each of which can be updated based on the error experienced on the corresponding movement and, through a coupling factor, from the error experienced on the reciprocal movement. This leads to an interaction that is controlled by the coupling factor whose value lies between 0 and 1, which determines the proportion of the error that each process uses to learn from the reciprocal movement. A coupling of 0 means that the Out and Return processes are independent whereas a coupling of 1 means that each process learns equally from errors in both movements. The coupling factor is dynamic, with its value depending on both the error experienced on the current trial, as well as the past experience of errors. Specifically, the model learns to associate errors on the Out and Return movements and then use the error on the current movement to anticipate whether a similar error is likely to be seen on the reciprocal movement. The more likely it is that a similar error will be observed, the higher the coupling will be on the current trial, so that the error on the next movement can be reduced.

**Fig 4:**
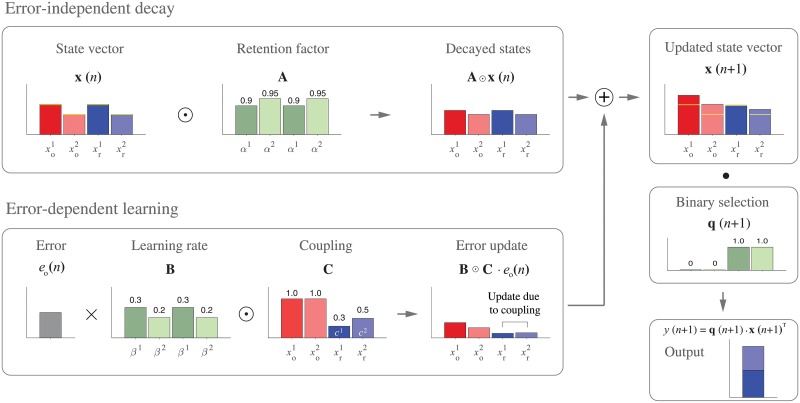
Model state update. **A**. The state update includes error-independent decay (top) and error-dependent learning (bottom). The four model states (top 1st plot) correspond to the two rates (superscripts, 1 and 2) and two movement directions (subscripts, o = Out and r = Return). The retention factor (top 2nd plot), which can be different for the two rates, determines the error-independent decay leading to decayed states (top 3rd plot). Error-dependent learning (bottom) is determined by the error experienced (*e*_*o*_(*n*) for an Out trial, bottom 1st plot), the learning rate, which can be different for the two adaptation rates (bottom 2nd plot), and a coupling factor (bottom 3rd plot). The coupling factor is always 1 for the states (both rates) corresponding to the current movement, but can have non-zero and different values for the states corresponding to the reciprocal movement. The error update (bottom 4th plot) is the product of the error, the learning rate and the coupling factor. The updated state vector (top far right) is the sum of the decayed states and error update (yellow bar shows the previous state vector). Finally, the motor output (bottom right plot) is the sum of the states corresponding to the the given movement as determined by the binary selection vector.

We used a state-space model that includes separate states for Out and Return movements and also two different rates of adaptation for each movement [16, 28]. This gives a total of 4 states (two directions and two rates) which we represent by the elements of a state vector (Fig 4, top left):

$$\begin{array}{c} x=[\begin{array}{cccc} x_{o}^{1} & x_{o}^{2} & x_{r}^{1} & x_{r}^{2} \end{array}], \end{array}$$ (2)

where subscripts represent the movement direction associated with the state (o = Out and r = Return) and superscripts represent the two rates (1 and 2). The motor output *y*(*n*) on each trial is the sum of the states for the particular movement direction, and is determined by the binary elements of the selection vector **q**(*n*) (Fig 4, right middle panel):

$$\begin{array}{c} y\left(n\right)=q\left(n\right)x{\left(n\right)}^{T} \end{array}$$ (3)

where **q**(*n*) = [1, 1, 0, 0] for Out movements and [0, 0, 1, 1] for Return movements. The error *e*(*n*) on each trial is the difference between the motor output and the task perturbation:

$$\begin{array}{c} e(n)=f(n)-y(n) \end{array}$$ (4)

where, *f*(*n*) represents the external perturbation (force field) and is either +1 (for the CW force field), –1 (for the CCW force field), or 0 (for the null field). Note that for channel trials the error is set to 0 by definition. After each trial, the state vector is updated as follows (Fig 4):

$$\begin{array}{c} x(n+1)=A⊙x(n)+B⊙C(n)\cdot e(n) \end{array}$$ (5)

where ⋅ is the multiplication of a scalar by scalar/matrix, ⊙ is element-wise multiplication, and **A** = [*α*^1^, *α*^2^, *α*^1^, *α*^2^] and **B** = [*β*^1^, *β*^2^, *β*^1^, *β*^2^] represent the retention factors and the learning rates, respectively, for the two adaptation rates. **C**(*n*) represents the coupling vector that determines how much of the error in the current trial should be used to update the states associated with the Out and Return movements. That is, **C**(*n*) = [1, 1, *c*^1^(*n*), *c*^2^(*n*)] and [*c*^1^(*n*), *c*^2^(*n*), 1, 1] for Out and Return movements, respectively, where *c*^1^(*n*) and *c*^2^(*n*) are the coupling factors for the two rates. Accordingly, on an Out trial, the Out process is fully updated (with a weight of 1), whereas the Return process is updated via the coupling factors *c*^1^(*n*) and *c*^2^(*n*). This is shown schematically in Fig 4, where an error from an Out trial is apportioned between the Out and Return states. In the following section, we describe how the coupling factor changes across trials as a function of the experienced errors.

#### The coupling factor

The coupling factors, *c*^1^(*n*) and *c*^2^(*n*), are essential parts of the model as they determine the interaction between the Out and Return processes for each adaptation rate. Each coupling factor can be the sum of a fixed component and an adaptive component:

$$\begin{array}{c} c^{i}\left(n\right)=c_{f}^{i}+c_{a}^{i}\left(n\right) \end{array}$$ (6)

where $c_{f}^{i}$ and $c_{a}^{i}\left(n\right)$ represent the fixed and adaptive components, respectively, and the superscript corresponds to the two adaptation rates.

In the model, the adaptive coupling is a function of errors that are experienced on each pair of Out and Return trials. When errors from the Out movements are repeatedly observed along with errors from the Return movements, an association (coupling) develops between these errors. The coupling is such that errors on one movement will lead to reducing the errors for the other movement. Therefore, when an error is experienced on the current trial, the associated states are updated for that movement direction, and the coupling factor also allows the reciprocal states to be partially updated to reduce the expected error on the subsequent trial.

**Fig 5:**
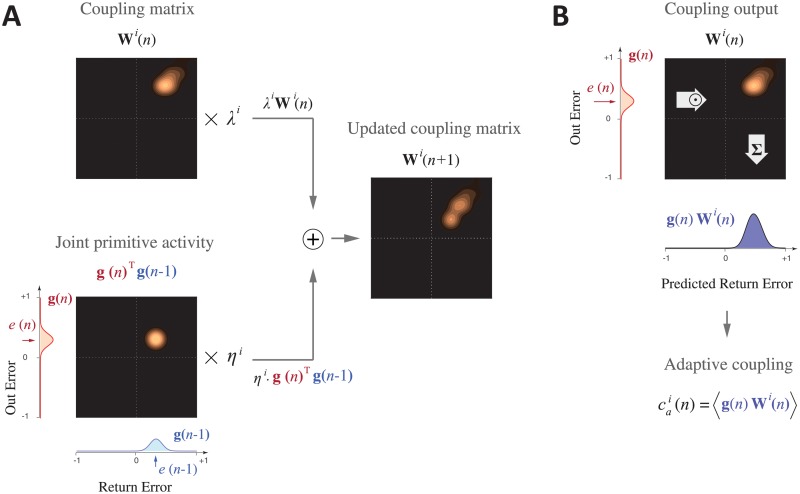
Model coupling. **A**. The update of the coupling matrix relates errors on Out and Return movements. As with the state update, the coupling matrix is updated by a combination of decay (top left) and an error-dependent accumulation process. The errors from the last two trials activate Gaussian tuning functions (**g**(*n*) and **g**(*n* − 1)) and the product gives a 2D isotropic Gaussian primitive in the coupling matrix (bottom left). The rate of decay and accumulation are determined by λ and *η*, respectively. There can be separate coupling matrices for the two adaptation rates. **B**. The final scalar coupling for a given trial is determined by multiplying the primitive for the current error by the coupling matrix and summing to determine the adaptive component of the coupling factor for that trial.

The errors in the Out and Return movements are each encoded by a set of primitives that form a two dimensional representation of the errors space. Specifically, the error space is uniformly tiled by a set of Gaussian tuning functions with the activity given by:

$$\begin{array}{c} \begin{array}{cc} g(n) & =[\begin{array}{c} g_{1}(e(n)),\ldots ,g_{m}(e(n)) \end{array}]\\ g_{j}(e(n)) & =\exp[\frac{-{\left({\bar{E}}_{j}-e\left(n\right)\right)}^{2}}{2\sigma^{2}}] \end{array} \end{array}$$ (7)

where, *m* is the number of primitives in each movement direction, ${\bar{E}}_{j}$ is the preferred error size for primitive *j* (which uniformly tile the error space), and *σ* is the width of each primitive. The pair of errors in the last two trials (*n* and *n* − 1) leads to an isotropic Gaussian activation (Fig 5A, bottom left plot) which is the joint activity of error primitives for the Out and Return movements **g**(*n*)^T^ **g**(*n* − 1).

Over the trials, this joint activity is accumulated and stored in a coupling matrix **W**^*i*^(*n*), whose elements represent the connection that is formed between the errors from the Out movements and the errors from the Return movements. There are potentially separate coupling matrices (superscript *i*) for each of the two adaptation rates. Fig 5A (top left plot) illustrates an example of the coupling matrix after several trials. As shown, the matrix represents the trace of errors from the Out and Return movements. For an Out movement on trial *n*, the coupling matrix is updated as follows (Fig 5A):

$$\begin{array}{c} W^{i}\left(n+1\right)=\lambda^{i}\cdot W^{i}\left(n\right)+\eta^{i}\cdot \text{sign}(e(n)\cdot e(n-1))\cdot g{\left(n\right)}^{T}g\left(n-1\right) \end{array}$$ (8)

where, λ^*i*^ is the retention factor and *η*^*i*^ is the accumulation rate of the coupling matrix across trials. The same update rule is applied for Return trials, except that the joint activity of primitives is transposed (that is, [**g**(*n*)^T^ **g**(*n* − 1)]^T^).

The sign of (*e*(*n*) ⋅ *e*(*n* − 1)) in the update Eq (8) determines whether the pair of Out and Return errors were of the same or the opposite signs. This appropriately adjusts the primitive contribution, which is only sensitive to the error magnitude, so as to predict the reciprocal error magnitude and sign.

Given the coupling matrix, we can determine the value of the adaptive coupling on each trial. For a given error on an Out trial with the pattern of activity **g**(*n*), the product **g**(*n*)**W**^*i*^(*n*) is an array whose elements represent the association that was formed between the observed error *e*(*n*) in the current movement, and all the possible errors that might occur in the reciprocal movement (Fig 5B). The average across the elements of this product determines the overall value of the adaptive coupling for the given trial:

$$\begin{array}{c} c_{a}^{i}\left(n\right)=\langle g\left(n\right)W^{i}\left(n\right)\rangle \end{array}$$ (9)

where, 〈〉 represents averaging. For Return movements, the same rule is applied except that the transpose of the coupling matrix is used (that is **W**^*i*^(*n*)^T^). Although we use **W**^*i*^(*n*) as a full matrix in the simulations and figures, the row and column sums are sufficient to simulate the model.

In summary, the pattern of activation **g**(*n*) caused by an error on a given movement is acted upon by the coupling matrix to determine how the observed error is associated with different possible errors in the reciprocal movement, and thus how much of the current error should be used to update the reciprocal process to reduce the possible errors for the next movement.

#### Model fitting

The full model has 11 free parameters, including the retention factors and the learning rates for each adaptation rate (*α*^*i*^, *β*^*i*^; 4 parameters), the fixed components of coupling ($c_{f}^{i}$; 2), the retention factors and accumulation rates for the coupling matrix (λ^*i*^, *η*^*i*^; 4), and the width of each primitive (*σ*; 1). Note that we do not constrain the relative values of the rate parameters (i.e., *α*^*i*^, *β*^*i*^) to exclusively represent fast or slow processes [28], but rather interpret these values from the fits as being associated with either fast or slow process. For the number of primitives (*m* in Eq 7), similar to previous models of sensorimotor learning (e.g., [31, 32]) we considered a fixed value (here, *m* = 10) for the model fitting and simulation procedures (the model fitting results were fairly robust in the range of *m* = 8 to 12).

We considered the full model and two reduced versions in which either the fixed or the adaptive coupling are set to zero. In addition, we consider models with separate coupling factors for the two adaptation rates (*c*^1^(*n*) and *c*^2^(*n*)), a shared coupling factor for both rates (*c*^1^(*n*) = *c*^2^(*n*)), or coupling for only one rate (*c*^2^(*n*) is set to 0). The combination of these different fixed and adaptive coupling factors (three) and coupling for the different rates (three) factorially gives 9 different variations of the model (M1 to M9), described in Table 1. In addition, we also considered the case in which there was no coupling, that is *c*^1^(*n*) = *c*^2^(*n*) = 0 (M10). In this case, the adaptive processes for the Out and Return movements are fully independent.

**Table 1:** Model details.

	Model coupling	Parameters in addition to *α*^1^, *β*^1^, *α*^2^, *β*^2^		DOF	Δ BIC	R^2^	% Best fit
		Fixed coupling	Adaptive coupling				
M1	Fixed for one rate	$c_{f}^{1}$	–	5	318.1	0.9473	0
M2	Fixed same both rates	$c_{f}^{1}=c_{f}^{2}$	–	5	274.5	0.9480	0
M3	Fixed separate each rate	$c_{f}^{1}$, $c_{f}^{2}$	–	6	256.0	0.9484	0
M4	Adaptive for one rate	–	λ^1^, *η*^1^, *σ*	7	12.5	0.9520	6.4
M5	Adaptive same both rates	–	λ^1^ = λ^2^, *η*^1^ = *η*^2^, *σ*	7	25.3	0.9518	7.4
M6	Adaptive separate each rate	–	λ^1^, λ^2^, *η*^1^, *η*^2^, *σ*	9	26.1	0.9520	0.33
M7	Mixed for one rate	$c_{f}^{1}$	λ^1^, *η*^1^, *σ*	8	16.2	0.9521	1.88
M8	Mixed same both rates	$c_{f}^{1}=c_{f}^{2}$	λ^1^ = λ^2^, *η*^1^ = *η*^2^, *σ*	8	0	0.9522	81.23
M9	Mixed separate each rate	$c_{f}^{1}$, $c_{f}^{2}$	λ^1^, λ^2^, *η*^1^, *η*^2^, *σ*	11	15.5	0.9524	2.76
M10	None	–	–	4	407.0	0.9458	0

We considered 10 model variants, which differed with regards to the fixed and adaptive coupling components, as shown. The basic uncoupled model (M10) had 4 parameters. Additional parameters were associated with the different variants of the models that included coupling. Goodness of fit measures for each model are also shown, in terms of *BIC* and *R*^2^. Note that Δ*BIC* indicates the *BIC* values for each model relative to the preferred model (M8 with Δ*BIC* = 0). The percentage best fit (% Best fit) indicates the percentage of bootstrap samples in which a given model showed the best fitting performance in terms of *BIC*.

Each model was fit to both the MPE and adaptation measures for all groups simultaneously. We chose to fit both MPE and adaptation measures so that the number of trials fit for all groups was the same (the number of adaptation trials varied between the groups depending on the paradigm, but the total number of trials including adaptation and MPE was the same for all groups). As the MPE and adaptation are in different units, we used an additional set of mapping parameters to linearly map these measures to the dimensionless model space (where error and adaptation range between -1 and 1). This adds four additional parameters to the model fitting process (one scaling and one offset parameter for each of the MPE and adaptation measures). After model fitting, the mapping parameters were used to transform the model predictions back to the experimental units for MPE and adaptation.

Model fitting was performed by minimizing the root mean square error between the model and the data (using *fmincon* in Matlab). Appropriate constraints were applied on the model parameters for optimization. Namely, the retention factors and the learning rates for both the state process (*α*^*i*^ and *β*^*i*^) and the coupling process (λ^*i*^ and *η*^*i*^), as well as the magnitude of the fixed coupling factor were constrained within the [0, 1] interval. The width of the Gaussian primitives was also constrained to be positive (i.e., *σ* > 0). When fitting each model to the average data or each bootstrap sample, the optimization was performed from multiple randomly selected initial points to avoid local minima.

Confidence intervals for the parameters of each model were generated by bootstrapping [33]. We generated 1000 samples, each of which was produced by randomly choosing 10 subjects (with replacement) from each group and averaging the data across subjects within each group. We then fit the models to the average data from all groups at once to obtain the parameters for that sample. The 95% confidence limits were taken as the 2.5 and 97.5 percentiles from the resulting distribution for each parameter across all samples.

The Bayesian Information Criterion (*BIC*) was used for model comparison:

$$\begin{array}{c} BIC=N\cdot \ln\left(\frac{SSE}{N}\right)+p\cdot \text{ln}\left(N\right) \end{array}$$ (10)

where, *N* is the number of trials, *SSE* is the sum of squared errors of the model fit, and *p* is the degrees of freedom of the model. The difference between the *BIC* values of two models represents half the log of the Bayes factor [34]. A *BIC* difference of greater than 4.6 (a Bayes factor of greater than 10) is considered to provide strong evidence in favour of the model with the lower *BIC* value [35].

After fitting all models to the average MPE and adaptation data, we calculated the difference in *BIC* between each model and the model with the smallest *BIC*. This provided a Δ*BIC* value for each model (which is 0 for the selected model).

Table 1 summarizes the *BIC* difference compared to the preferred model, M8. As shown, the model with no coupling (M10) shows the poorest performance in explaining the data. The models with fixed coupling (M1 to M3) are also far from the best model with the *BIC* difference of more than 250. However, models that include adaptive coupling (either adaptive coupling only or a mixture of adaptive and fixed coupling; M4 to M9) show substantial improvement in fitting the data. Among these models, M8, which includes mixed coupling shared between two adaptive rates, shows the best performance in terms of *BIC*, and accounts for more than 81% of the bootstrap samples as the best model. Note that although the *R*^2^ values are similar across all models, this measure is independent of the number of data points and the degrees of freedom (parameters) in a model. In contrast, the *BIC* values scale with both the number of data points and the degrees of freedom. As such, small improvements in *R*^2^ for a model can translate into a large difference in the likelihood and hence the *BIC*.

**Fig 6:**
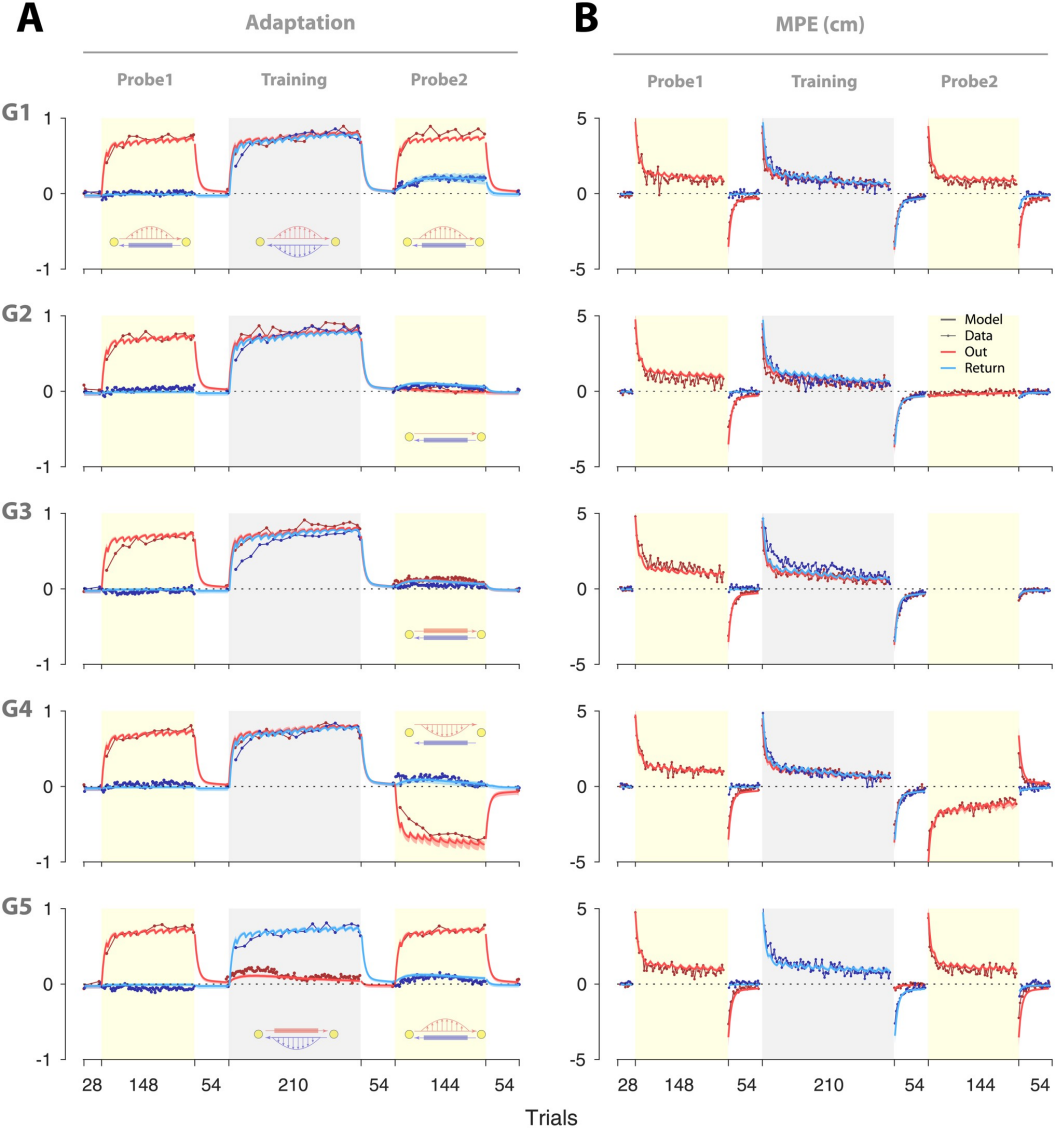
Model fits across three phases for the five groups. Fits of the selected model (M8) to adaptation (**A**) and MPE (**B**). The model was fit to both measures (adaptation and MPE) concurrently for all groups. The solid lines show model fits to group data (red lines for Out and blue lines for Return movements) and the error bars (shading) represent the 95% confidence intervals on model fits from bootstrapping.

**Fig 7:**
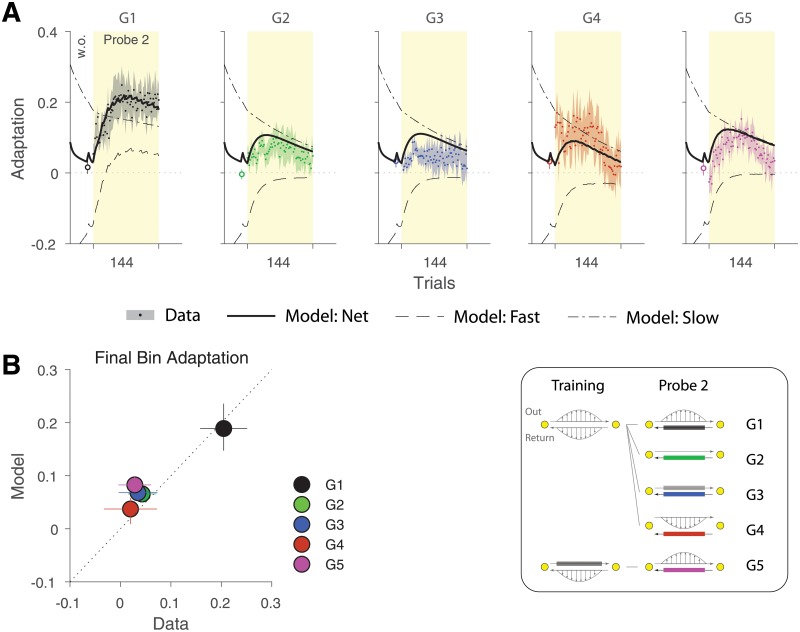
Model fits for Probe2. **A**. The net model output (solid black line) as well as the fast (dashed line) and slow (dash-dotted line) adaptive states shown for the Return trials of Probe2 for all groups. At the end of the washout period prior to Probe2, the fast and slow processes are at opposite sides, predicting a rebound during Probe2 for all groups. The extent of this rebound is larger for G1, due to greater increase of the fast process and slower decay of the slow process compared to other groups. **B**. Adaptation in the final 12 trials of Probe2 (i.e., final bin as in Fig 3C). The model prediction is plotted against the data. The error bars in the horizontal dimension represent the standard error of the mean across subjects, and the error bars for the vertical dimension are the 95% confidence intervals from bootstrapping.

Fig 6 shows the model fits from the selected model (M8; parameters in Table 2) for adaptation and MPE measures across all groups. Note that for the Probe1 and the Training phases, the model fits of G1 are identical to that of G2 to G4 (due to identical trial sequence in these groups), and very similar to G5. The main difference in model fits across groups, therefore, is expressed in the Probe2 phase. To better examine the model behaviour during Return trials of Probe2, Fig 7A illustrates the net model output (solid black line) as well as the fast (dashed line) and slow (dash-dotted line) adaptive processes associated with the Return movements of Probe2 for each group. Importantly, although the net adaptation is close to the baseline for all groups before the onset of Probe2, the fast and slow processes are not fully washed out, but rather take nonzero values in opposite directions (positive for the slow and negative for the fast process). This means that a rebound of adaptation is expected during Probe2 for all groups as seen in Fig 7A. For G1, however, the amount of rebound is different from the other groups.

**Table 2:** Best fit parameter values for M8 with 95% confidence intervals.

Parameters	Best fit value	Lower bound	Upper bound
*α*^1^	0.948	0.941	0.953
*β*^1^	0.228	0.220	0.260
*α*^2^	0.995	0.993	0.995
*β*^2^	0.037	0.028	0.045
$c_{f}^{1}=c_{f}^{2}$	0.023	0.005	0.043
λ^1^ = λ^2^	0.995	0.990	0.997
*η*^1^ = *η*^2^	0.089	0.060	0.112
*σ*	0.058	0.043	0.078

As shown, the fast process in this group increases beyond the level observed in other groups, and the slow process shows a slower decay in the course of Probe2 phase. As a result, the net adaptation increases to a higher extent (due to the larger rebound of the fast process) and persists longer (due to slower decay of the slow process) by the end of the phase. This is explained in the model by coupling of adaptation that partially transfers learning (or error signals) from the Out movements to the Return movements for both fast and slow processes. The outcome, therefore, is expressed as a relatively larger increase in the net adaptation beyond the expected spontaneous recovery that is observed in other groups.

Note that the parameters for the fast and slow processes are the same for all groups, as the model is fit to all groups at once (i.e., the parameters *α*^1,2^ and *β*^1,2^ in Table 2). These parameters are mainly constrained by the observed spontaneous recovery (e.g., G2 and G3 in Fig 7A) and the potential savings [28] during adaptation (e.g., faster relearning for the Out movements during the Training phase; Fig 2, G1 to G4). For example, larger savings could be reflected in a larger retention factor and a smaller learning rate for the slow process. Similarly, a faster rebound in spontaneous recovery could be reflected in a smaller retention factor and a larger learning rate for the fast process. What makes the difference in the Probe2 behaviour between G1 and other groups (i.e., Fig 7A) is the effect of coupling on the fast and slow processes. That is, although all groups share the same fast and slow rate parameters, the coupling effect in G1 leads to transfer of error signals from Out to Return movements, which then elevates the adaptation in both fast and slow processes compared to other groups.

The model fits in G1 (Fig 7A) also show a slight, yet progressive decay of net adaptation towards the end of Probe2 phase. This is captured by the retention factor for the coupling matrix (λ^1^ = λ^2^ = 0.995; Table 2), indicating that the coupling decays over time, leading to progressive reduction of transferred adaptation. Finally, we examined the model fits for adaptation in the final bin of Probe2 phase (as in Fig 3C). Fig 7B illustrates the adaptation from the model fits plotted against the adaptation data for each group. As shown, the model appropriately captures the final level of adaptation across different groups, with higher adaptation for G1 and lower adaptation for other groups.

In order to understand how the selected model captures the experimental data, we focus on changes in coupling across trials for each group. The coupling factor represents the amount of learning that is transferred from the current movement direction to the reciprocal direction. The fixed component of the coupling ($c_{f}^{i}=0.023$ for M8) represents the baseline interaction (generalization) which was small. This value captures the limited transfer of learning from the Out movements to Return movements in Probe1. In contrast, the adaptive component of the coupling plays the major role in explaining the difference between the groups in Probe2.

**Fig 8:**
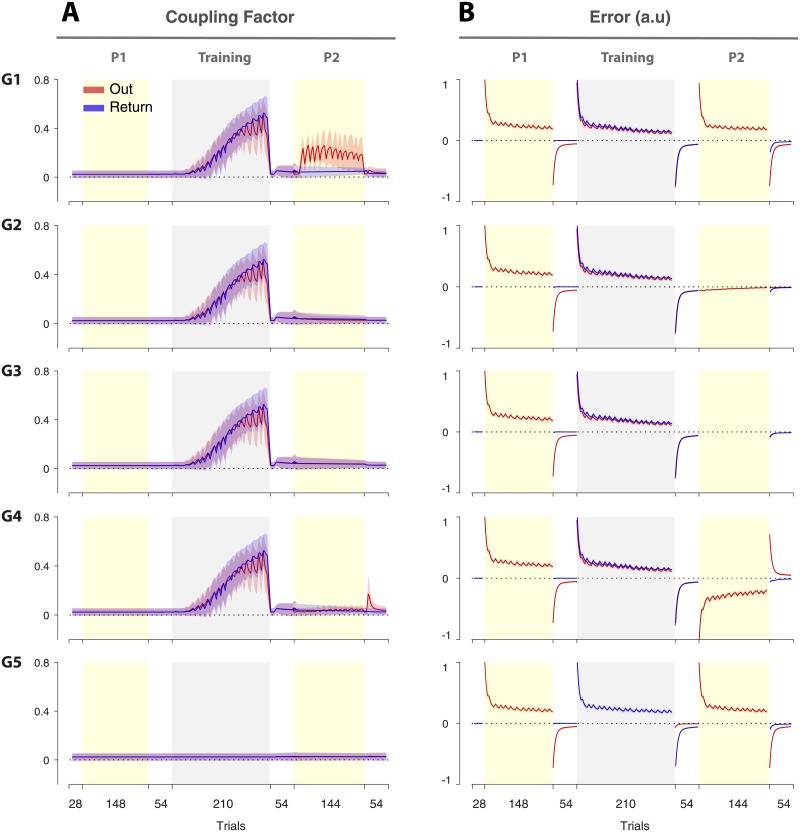
The coupling factor across three phases for the five groups. **A**. The trial-series for the coupling factor (model M8) shown for all five groups for the Out (red) and Return (blue) movements. The error bars represent the 95% confidence intervals, obtained by bootstrapping. **B**. The trial-series for error from the model for the Out (red) and Return (blue) movements.

In Fig 8A the time course of the coupling factor (mixture of fixed and adaptive) for M8 is illustrated for both the Out and Return trials. This shows the coupling expressed on each trial which is determined by the memory trace **W**(*n*) and the current error. As shown, the value of the coupling factor during Probe1 is small, reflecting only the fixed component of coupling (0.023). In the Training phase, for G1 to G4, the concurrent force field exposure caused errors for both movement directions. This led to the formation of adaptive coupling which increased the overall coupling to 0.53 by the end of the Training phase. Note that in the beginning of the Training phase, the coupling factor shows a slow growth due to the rapid reduction of error in the first few trials so that the same pair of errors are infrequently experienced. However, in the following trials, as exposure to the force field progresses, the errors change more slowly. As such, the same errors are repeated between the Out and Return movements for a longer period (Fig 8B). This activates the same primitives repeatedly, causing a stronger association between the corresponding error values from each movement direction.

During Probe2, the adaptive coupling was high during the Out trials of G1, but remained close to baseline for the other groups. Crucially, subjects in G1 experienced exposure trials in the Out movements of Probe2, which resulted in similar errors that were previously experienced during the Training phase. Therefore, the coupling expressed in response to these errors was high during Probe2. As shown in Fig 8A, G1, the value of the coupling factor is around 0.15 throughout Probe2. This causes an increase in adaptation during the Return trials of Probe2 in G1 (Fig 3A).

In G2 and G3, the Out trials are, respectively, null and channel trials, neither resulting in errors that would induce coupling (Fig 8B, G2 and G3). In G4, subjects experienced a reverse force field during Probe2, which resulted in errors that were in the opposite direction to the ones experienced during the Training phase (Fig 8B, G4). Since these errors were not coupled previously, the coupling also remains low in this group. Finally, in G5, subjects never experienced concurrent errors throughout the experiment, and thus no adaptive coupling was developed in this group.

**Fig 9:**
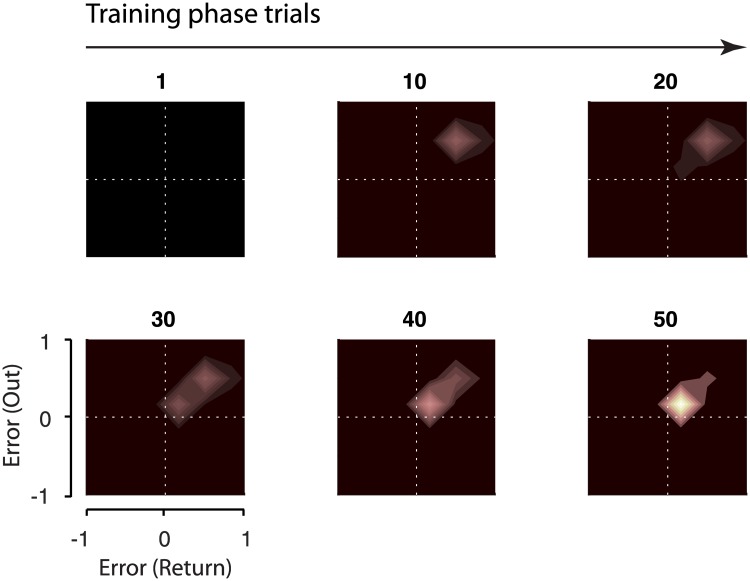
The coupling matrix. The coupling matrix for model M8 is shown for selected trials during the Training phase experienced by G1 to G4 (Training phase was identical for these groups). Before experiencing any errors, all elements of the matrix are zero (Trial 1). The force field is then experienced on both Out and Return movements, resulting in the development of coupling between the corresponding errors (both positive errors). Early in Training phase (trials 10 and 20), there are large errors and the coupling matrix accumulates a weighting away from the origin. As adaptation progresses (trials 30 to 50) the errors reduce. As a result, the coupling shifts towards the origin and increases. The coupling matrix for G5 (not shown) remains unchanged (as in Trial 1) throughout the Training phase as errors are only experienced on the Out movements.

Fig 9 shows the coupling matrix for a few selected trials from the Training phase for G1 to G4. In these groups, concurrent adaptation during the Training phase leads to associations forming between the errors from the Out and Return movements. Since the coupling matrix accumulates the joint activity of primitives on each pair of Out and Return trials, the more a particular pair of errors is repeated, the stronger the association will be in the coupling matrix. Therefore, for the early trials of the Training phase where the errors reduce rapidly (Fig 9, trials 10 and 20), there is weaker coupling formed between the corresponding errors. Whereas in the later trials, where the error plateaus to small values, the coupling shifts towards the origin and accumulates to a larger extent (Fig 9, trials 30 to 50).

## Discussion

We examined the coupling of learning between motor memories associated with reciprocating Out and Return movements when adapting to novel force field dynamics. Subjects initially experienced a force field for one movement direction (Out) while the reciprocal direction (Return) was performed in a channel. Consistent with previous studies [7, 9, 36], we found minimal coupling of learning in this case in that adaptation to force field for one movement direction (Out) did not lead to adaptation for the opposite movement direction (Return). More importantly, we found that coupling was not fixed but could develop if the force field was experienced concurrently for both Out and Return movements. This coupling was specific to the sign of errors experienced in each movement direction, and required that the errors for both movements be experienced consecutively rather than in separate phases of the experiment. A context-dependent state-space model was developed in which coupling was an adaptive process, relying on a coupling matrix, which formed a memory trace of the consecutive errors that were experienced in Out and Return movements.

The effect of coupling was examined by contrasting the adaptation behaviour across groups during the second Probe phase. Coupling was reflected by an increasing adaptation for the the Return channel trials. This was only seen for G1 who had experienced the force field for both Out and Return movements concurrently. Unlike the other groups, this group showed an increase in adaptation above what would be expected based on spontaneous recovery alone. That is, due to relatively short washout period between the Training phase and Probe2, adaptation rebound happened, to some extent, for all groups. This rebound was explained by the dual-rate property of the model [28], where slow and fast processes were not fully washed out by the onset of the second Probe, (Fig 7A), but rather maintained values of opposite sign which then led to spontaneous recovery of the net adaptation. This was reflected in the overall level of adaptation throughout the Probe2 phase (averaged across trial bins), which was larger for G1 compared to other groups (Fig 3). Moreover, adaptation in G1 persisted longer so that by the end of the phase (final bin), adaptation was larger for G1 than other groups. These results suggest that the enhanced coupling between Out and Return movements in G1 further raised the adaptation level for Return movements.

The clear distinction between the adaptation behaviour in G1 and that of the other groups indicates that coupling is enhanced and revealed under specific conditions. That is, it may require concurrent adaptation in the Training phase, and the re-exposure to previously experienced errors during the Probe2 phase. However, a considerable rebound was also observed in the Probe2 phase for G4 and G5 (i.e., Fig 3B), which, although not significantly different from the spontaneous recovery observed in G2 and G3, may reflect nonspecific effects of sudden exposure to force field in the Out movements of Probe2. When the re-exposure occurs, regardless of the force field direction, the consequent errors might trigger a retrieval of previous adaptation for the Return movements (i.e., from the Training phase) which leads to expression of adaptation as a strong rebound in the Probe2 phase. Such instant recall of adaptation in the Return movements may not be due to coupling (i.e., transfer of learning from Out movements) for, unlike in G1, it decays quickly back to the baseline. But it may reflect retrieval of previously successful commands due to sudden lack of reinforcement or success (i.e., experiencing errors; [30]). Our proposed model, in the current form, does not account for such nonspecific effects. Indeed, further work is required to better understand the mechanisms that underlie such effects.

In our proposed model, the enhanced generalization observed for G1 is attributed to a coupling matrix that keeps track of the errors that occurred consecutively for Out and Return movements. When these errors are revisited later for one movement (Out), they prompt a retrieval of errors in the reciprocal movement (Return), which then leads to increased transfer of learning (coupling) to reduce the effects of such expected errors. This interpretation is consistent with the studies suggesting that the sensorimotor system keeps a memory of previously experienced errors [31, 37]. However, a more parsimonious interpretation would be that the enhanced generalization observed in G1 is based on model-free mechanisms of learning [38–40]. As such, when subjects are re-exposed to force field in the Out movements of Probe2 phase, they retrieve the memory of previous successful actions that was formed during the Probe1 and the Training phase. This relearning based on previous successful actions might then generalize across movement directions more broadly than the error-based learning in the initial adaptation (i.e., Probe1). This hypothesis relies on two assumptions. First, that the initial and the secondary learning phases are governed by distinct learning mechanisms, with the former mainly based on error-dependent processes and the latter on the basis of recalling previous successful actions. Second, that these mechanisms might generalize across contexts differently. There is an ongoing debate regarding the first assumption [31, 37, 39, 40], however, it has been suggested that generalization of model-free learning based on reinforcement signals contributes only locally and to a limited extent to generalization [41, 42].

The coupling we observed might also arise from cognitive processes associated with adaptation [43–45]. That is, when subjects experience a concurrent force field perturbation for Out and Return movements (i.e., the Training phase in G1 to G4), a form of statistical learning might be involved indicating that the probability of a force field exposure for a Return movement, given the force field in the Out movement, is essentially one. This could lead to a decision-making policy in which an adapted movement plan for a Return movement is reinstated, every time a force field is experienced in the Out movement (i.e., Probe2 for G1). Indeed, the direction of the Out force field could act as a salient cue that activates such a movement plan for G1, but not for G4. This interpretation would be consistent with the failure to reinstate Return adaptation for G5, as no concurrent exposure is experienced in this group, and hence no such a policy can be formed.

Coupling might also involve explicit and/or implicit mechanisms [43, 46] in learning. Previous studies have suggested that explicit and implicit mechanisms could be represented, respectively, by fast and slow learning processes [47]. In our modelling, we examined several cases where coupling affects fast and slow processes in different ways. For instance, coupling could affect only one of the processes (i.e., M1, M4 and M7; Table 1), or both processes equally (M2, M5 and M8), or each process separately (M3, M6 and M9). The model fitting results suggested that a model, in which both fast and slow processes are equally involved in coupling, could describe the data best (i.e., M8). Given the possible relation between fast/slow and explicit/implicit representation of learning, our results could imply that explicit and implicit mechanisms might be involved in coupling to the same extent.

Our proposed model is, in essence, consistent with previous context-dependent state-space models of sensorimotor learning, but also provides further insight as to how context-dependent motor memories might interact. For example, the generalization of learning across contexts in previous models is typically captured by a context-dependent learning rate, which assumes a fixed amount of adaptation transfer between two given contexts (e.g., movement directions; [9, 16–18, 20, 48]). In our model, such context-dependent learning rate exists in the form of a coupling factor, except that it is not fixed, but is rather adaptive and can increase depending on the experienced errors for each movement direction. This adaptive mechanism relies on the interaction of error-based primitives that encode errors in Out and Return movements, and whose joint activity is accumulated in a coupling matrix as how strongly errors from Out and Return movements are coupled. Such a mechanism is also consistent with the notion of associative (Hebbian) learning [49], wherein the motor system learns an association between the observed stimuli in different conditions. As such, experiencing errors in both contexts consistently causes repeated activation of corresponding primitives, which then leads to an increase in the connection weights between those primitives. As a result, when an error is experienced in one context (for example, the Out movements), it not only updates the motor memory of the corresponding context, but also partially modifies the memory of the alternative context (the Return movements).

Similar error-based primitives have also been used previously to account for environmental consistency in sensorimotor learning [31, 32]. In these models, a similar adaptive mechanism, based on error-dependent primitives, determines the learning rate to adapt to the environmental variations. As such, the learning rate decreases for highly variable environments and increases for more consistent environments. These models, however, mainly focus on the learning rate within a single context. Here, we extend the notion of adaptive learning to the coupling of motor memories across contexts. That is, how learning under one movement context should affect the adaptive behaviour in other contexts.

The notion of coupling is defined based on accumulating a history of experienced errors occurred consecutively between Out and Return movements. This is in accord with previous studies suggesting that a history of previously experienced errors determines the learning behaviour [31, 37]. For example, it has been shown that the rate of learning is increased for errors that are repeatedly experienced in the past, but not for the new errors [31, 37]. In line with these studies, an interesting prediction for the adaptive coupling model would be that if subjects concurrently adapt to a gradually imposed force field in both Out and Return movements (i.e., in Training), such that the experienced errors remain small, the coupling would only form for very small errors between Out and Return movements. As a result, only small errors would be allowed to transfer between movement directions via coupling, but not the large errors. Such modulation of coupling by the error size requires further investigation as a future work.

Previous findings have shown that motor memories are protected, with a small learning rate and high retention, for contexts that are distant from the current active context [18]. This allows the sensorimotor system to preserve multiple memories for different contexts and prevent them from changing due to irrelevant stimuli (error signals). As such, it might seem detrimental for the motor system to allow changes in the memory of a distant, non-active context based on the errors observed in an active context. However, we suggest that under specific conditions, it could be beneficial to have flexible interaction patterns (coupling) between context-dependent motor memories, and increase such interaction when needed. For example, when the task environment alternates between two different contexts, each associated with a particular external perturbation, the motor system can learn that an error in one movement context could be predictive of an error in the following, reciprocal context. Therefore, the observed error could be used both to improve the performance in the corresponding context and to modify the behaviour for the following context so as to reduce potential future errors.

Coupling might also influence retention. It has been suggested that, in the absence of error, learning decays faster for the current active context, while is retained for distant nonactive contexts [18]. One potential way that coupling might influence memory decay could be to increase the decay of learning for distant contexts as a result of decay in the current context. In our model, we assumed the same amount of decay for both Out and Return contexts, and focused on the coupling effects on the learning rate. Further work is required to examine the mechanisms by which coupling affects memory retention.

Our results suggest a flexible interaction between context-dependent motor memories that allows for sharing information (error signals) among different contexts to efficiently improve overall performance.

## Supporting information

S1 Data Adaptation and kinematic error data. Data is stored in a Matlab table with the following columns: ‘TrialNumber’ indicating the list of trials, ‘Out_Ret_Trials’ indicating the Out (1) and Return (2) trial numbers, ‘PostBreakTrials’ specifying the trails that immediately followed a rest break (these trial were excluded from analysis), ‘Field’ indicating the trial type (0 = Null, 1 = Force field, 2 = Channel), ‘Adaptation’ indicating the adaptation measure for channel trials, and ‘KinematicError’ indicating the max perpendicular error (MPE) for non-channel trials.
(MAT)

S2 Data Data information. A Matlab script containing details about the data table.
(M) Click here for additional data file.
